# Bilobed spleen: an extremely rare imaging finding

**DOI:** 10.1259/bjrcr.20170021

**Published:** 2017-08-07

**Authors:** Hosameldeen Mostafa Ali

**Affiliations:** Radiology Department, Benha University, Benha, Egypt

## Abstract

The spleen has a wide range of congenital anomalies regarding its shape, location, number and size. Unsuspected splenic congenital anomalies are commonly depicted on sonography orCT and sometimes pose a diagnostic dilemma for the radiologists and clinicians. The bilobed spleen is an extremely rarely encountered condition. It is most commonly detected during abdominal surgeries. The bilobed spleen is usually bigger in size than the normal spleen, so it is more predisposed to trauma. In this case report, we have presented a case of the bilobed spleen, which was incidentally suspected during abdominal ultrasonography of an 11-year-old girl complained of recurrent left hypochondrium pain. The bilobed configuration is confirmed with CT examination of the abdomen.

## Background

The spleen is a large encapsulated organ mainly encompassing vascular and lymphoid tissue, situated in the upper left quadrant of the abdominal cavity between the diaphragm and fundus of the stomach and related to the tail of the pancreas and the upper pole of left kidney. The spleen serves to generate immune reactions and eliminates the foreign substances and aged erythrocytes from the blood stream. Spleen is a widely variable organ regarding its size, shape, fissures and position. Some of its variations might form a diagnostic challenge and could result in imaging pitfalls and misinterpreted as a pathological process. Most of the splenic pathology and morphologic anomalies are often clinically silent. Therefore, it is important to make a careful evaluation of the splenic parenchyma, rather than just undertake a routine measurement of splenic size, to avoid missing significant pathology and morphologic anomalies. We report here an extremely rare case of bilobed spleen with medial (internal) and lateral (external) lobes connected at the splenic hilum.

## Discussion

Relatively little published about the spleen in the imaging literature. The exact functions of the spleen are still somewhat of a mystery. In comparison to other abdominal organs, there is a significant paucity of scientific investigations involving the spleen. The spleen has been named “the forgotten organ”. Careful examination of the spleen can bring important diagnostic clues in congenital anomalies and pathologies such as oncologic, haematologic, infectiousmetabolic, abdominal trauma, portal hypertension and many other focal or diffuse splenic changes of different aetiologies.

The normal position of the spleen lies in the left upper quadrant along the shaft of the 10th rib. The spleen is the only alimentary tract structure that does not develop directly from the gut. The position of the spleen is in part maintained by several suspensory ligaments, including gastrosplenic, lienophrenic, lienocolic and lienorenal ligaments.^1^

The variability in the shape, notches and fissures has been explained on the developmental basis of the spleen. Spleen has its origin from the mesenchyme of dorsal mesogastrium, which lies over the dorsal pancreatic endoderm, as a long strip of cells next to the developing stomach. Cells required for the haemopoietic function arise from the yolk sac wall, near the dorsal aorta. Splenic vascularization arises initially by branches from the dorsal aorta. The haematopoietic function is lost with embryo development. Lymphoid precursor cells migrate into it late in foetal life from the central lymph organs. The earlier lobulated structure of the spleen disappears but is indicated by the presence of notches on the upper border of the adult.^2^

Typically, the spleen is a single organ, but it is common for smaller amounts of splenic tissue (splenunculi or accessory spleens) to surround the main body, particularly close to the pancreatic tail. Usually, these are single, but sometimes a few are present. Splenunculi are characteristically rounded, smooth-walled masses, most often 1–2 cm (but can be larger), that are located in the proximity of the spleen (usually the splenic hilum). They typically demonstrate a similar density to the spleen, whether on contrast-enhanced or non-contrast imaging, which usually differentiates them from lymphadenopathy or peritoneal and omental masses.^3^

Advances in imaging techniques are expected to detect splenunculi more frequently increasing the importance of being able to differentiate these lesions from more sinister pathologies.^4^ It should be distinguished from enlarged lymph nodes, as it follows the exact density and enhancement of the spleen on images obtained with various imaging phases.^5^

The detection and characterization of the rare bilobed spleen are of critical importance. The bilobed spleen may mimic lymphadenopathy and tumours in adjacent abdominal organs, such as the pancreas, the left adrenal gland and the left kidney. The medial (internal) lobe of the bilobed spleen should be differentiated from hypervascular pancreatic tumours like neuroendocrine tumours. Similarly, the medial splenic lobe should be differentiated from metastatic lesions or lymphadenopathy in the splenic hilum when they enhance the same degree as the spleen.^6^

The bilobed spleen should also be differentiated from other benign splenic variants like splenunculus. Accessory spleens, also known as supernumerary spleens, or splenunculi or splenunculus, are congenital foci of healthy splenic tissue that is separate from the main body of the spleen.^7^

The medial (internal) splenic lobe of the bilobed spleen intimately connected to the splenic hilum unlike other benign splenic morphologic variants like splenunculus or splenosis, which are separate from the splenic hilum. It could extend medially to displace and compress the pancreatic tail and the upper pole of left kidney with concomitant unexistent lienorenal ligament. It could indent the posterior aspect of gastric fundus mimicking iceberg tumour.^8^

**Figure 1.**
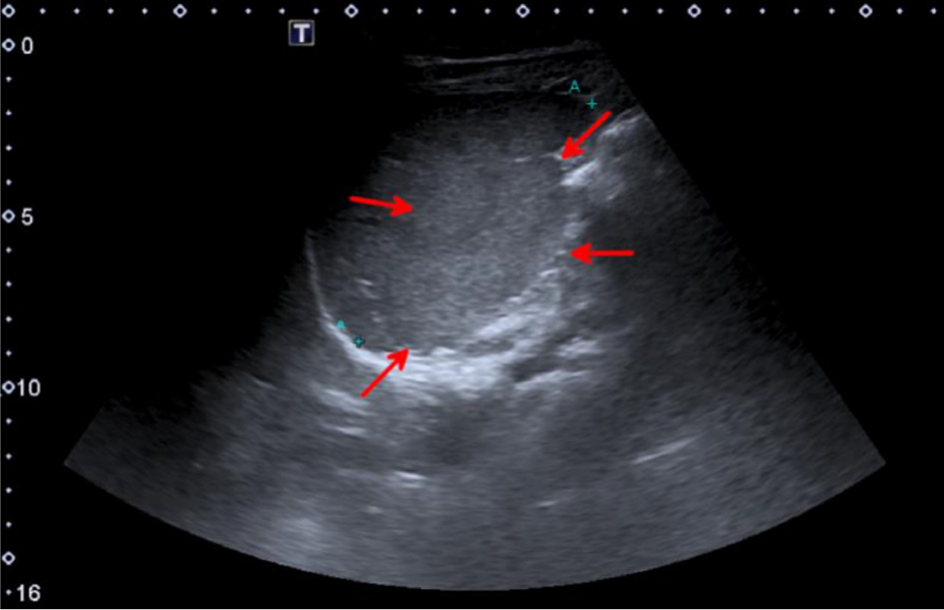
Long axis ultrasonography image of the spleen. The medial (internal) lobe of the spleen (red arrows) seen as a smooth ill-defined bulge from the splenic hilum. The medial lobe isoechoic to the rest of the spleen.

US and CT remain the major modalities used to visualize the spleen parenchyma. The normal spleen density is equal or less than the density of normal liver on CT images. The use of iodinated contrast agents improves tissue contrast. The US is a non-invasive, highly sensitive and specific imaging technique for the evaluation of splenic size and anomalies^9^ (Figure 1).

The spleen is usually easy to evaluate with ultrasound via an intercostal approach in suspended respiration; positioning the patient in the lateral decubitus position (right side down) may improve visualization. Normal splenic parenchyma is homogeneous and more echogenic than both the liver and kidney.

**Figure 2.**
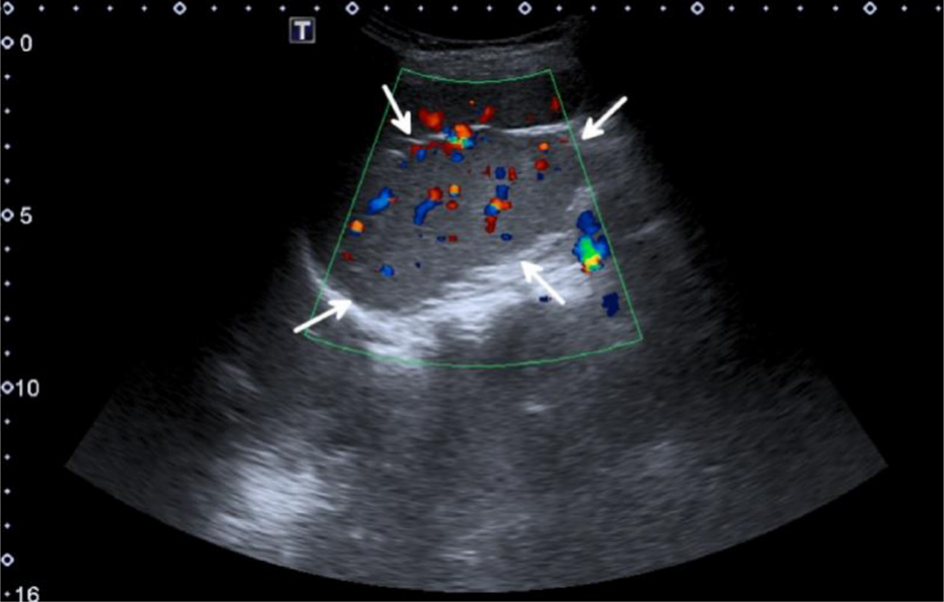
Long axis colour Doppler ultrasonography image of the spleen. The medial (internal) lobe of the spleen (white arrows) seen as a smooth ill-defined bulge from the splenic hilum. The medial splenic lobe demonstrated enriched vascularity like the rest of splenic parenchyma.

The spleen shows a variety of pathological entities. The US is the first imaging modality performed for its assessment. When it is not conclusive, the addition of intravenous contrast agents improves performance and aids in narrowing the differential diagnosis. Benign entities include cysts, accessory spleen, infarcts, abscess, benign masses and trauma. In most of these cases, contrast-enhanced ultrasonography (CEUS) can reach a diagnosis. Malignant entities include lymphoma and metastases, where CEUS is also useful with some limitations. The technique is performed easily, fast, with no radiation, even in patients suffering renal insufficiency^10^ (Figure 2).

CEUS is useful in cases where there is doubt over the origin of a parasplenic mass. Characterization of an accessory spleen (splenunculus) or of tissue that has arisen as post-splenectomy or trauma (splenosis) is aided by CEUS. The medial (internal) splenic lobe is found alongside the normal spleen in the left upper quadrant. It could reach a large size equal or even larger than in the lateral (external) lobe. It is wedge-shaped and isoechoic with adjacent splenic parenchyma. A pedicle of flow from the splenic artery may be demonstrated with colour Doppler ultrasound. In most cases, the diagnosis is straightforward and CEUS is not required; however, large or atypically located medial splenic lobe can cause diagnostic uncertainty.^11^

CEUS can confirm that a mass represents ectopic splenic tissue for both splenunculi and medial splenic lobe by demonstrating an enhancement pattern typical of a normal spleen. A mottled pattern may be seen in the parenchymal phase but, most importantly, the tissue will display persistent late-phase enhancement, differentiating the mass from other lesions such as pancreatic tail tumours, splenic hilar lymph nodes, adrenal lesions, gastric fundus masses and metastatic deposits, which do not have the characteristic of sequestrating contrast microbubbles and will show late-phase contrast washout.^12^

We suggested CEUS of the spleen, but the ultrasound contrast agent is not approved by the local health authorities in the United Arab Emirates. The child’s guardian (father) and clinician refused and preferred to carry out the MR examination of the abdomen instead to define the relations of the mass. The clinician did not concur that the mass was a medial splenic lobe and considered it a space occupying lesions arising from either gastric funds or tail of the pancreas.

CT remains the most useful preoperative investigation to measure splenic volume, to exclude lymph nodes or masses at the splenic hilum, and to detect and characterize accessory spleens, splenic abscesses, splenic focal lesions and bilobed spleen.

On unenhanced CT scans, the spleen has an attenuation similar to that of the liver, approximately 40 Hounsfield units. Normally, the liver and spleen densities are within 25 H on dynamic contrast-enhanced CT scans^13^ (Figures 3–11).

**igure 3.**
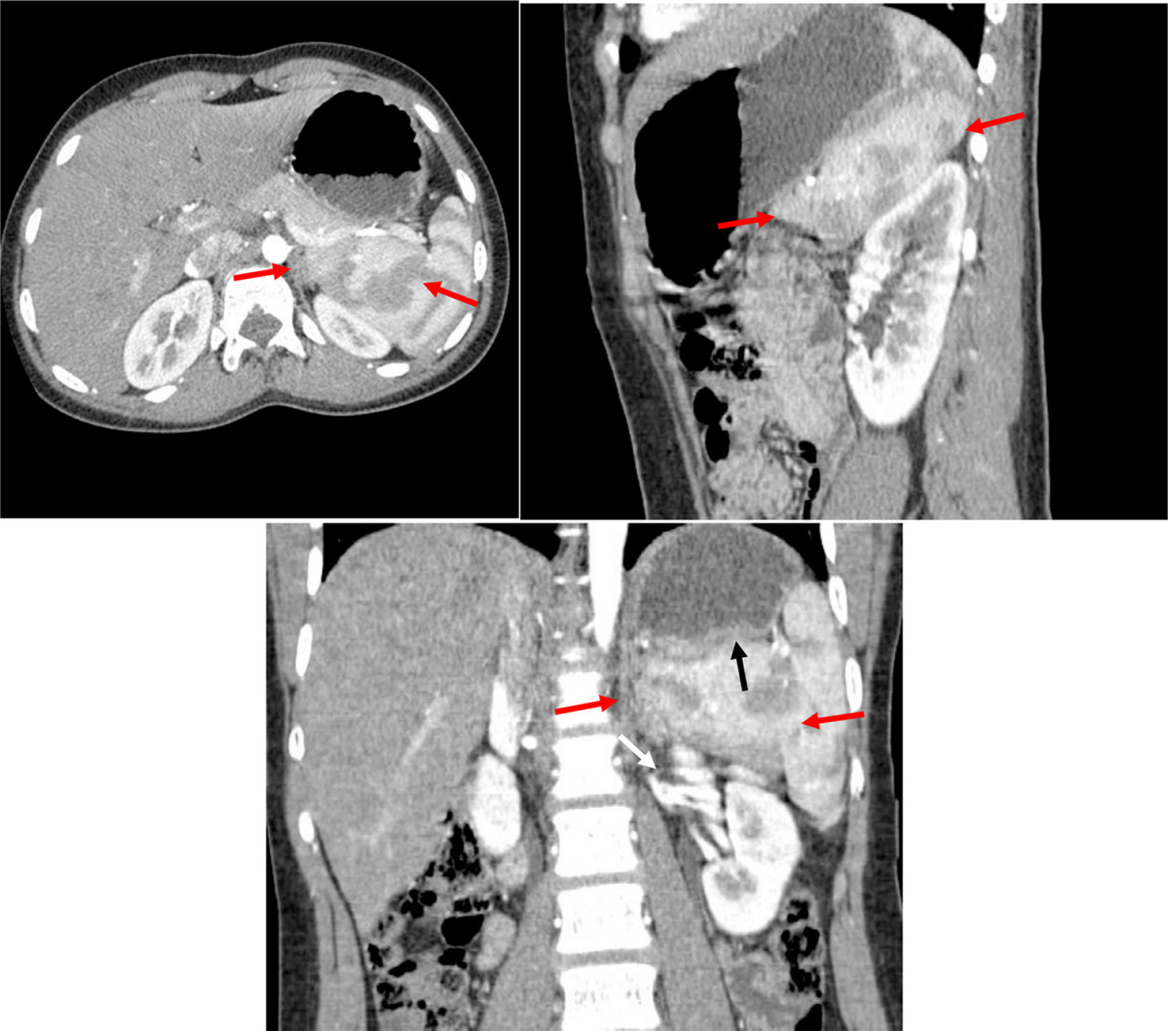
Concentric axial, sagittal and coronal multiplanar reformations contrast-enhanced CT images acquired during the arterial phase through the mid-spleen demonstrates the medial (internal) lobe of the spleen (red arrows) shows homogeneous CT density and isodense to the reminder of the spleen. The medial (internal) lobe of the spleen extends medially displacing the upper pole of the left kidney (white arrow) downwards and gently indenting the posterior aspect of the gastric fundus (black arrow). Both the medial (internal) and lateral (external) splenic lobes show mottled (zebra lines) pattern.

**Figure 4.**
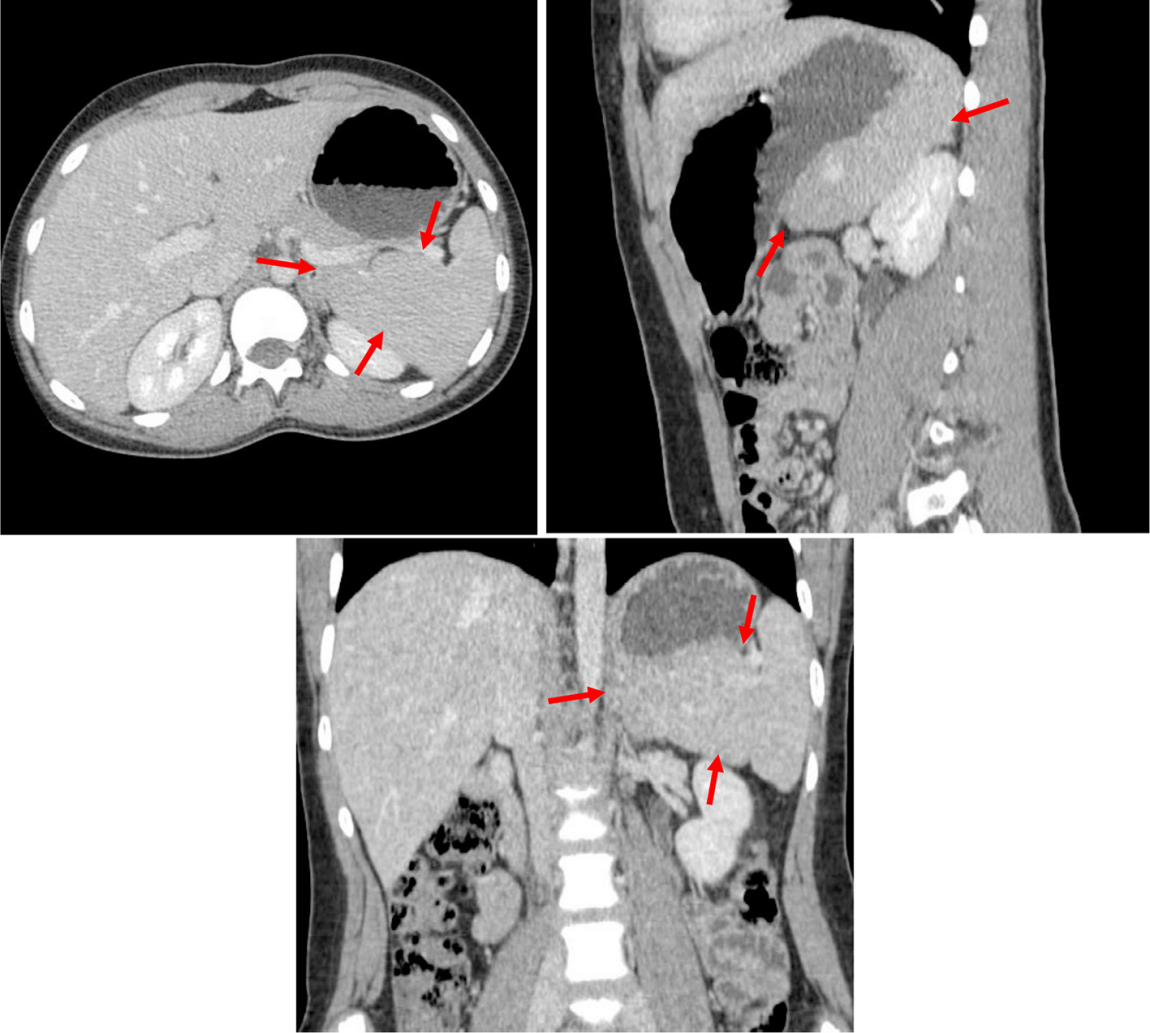
Concentric axial, sagittal and coronal multiplanar reformations contrast-enhanced CT images acquired during the portal venous phase through the mid-spleen demonstrates the medial internal lobe of the spleen (red arrows) shows homogeneous CT density and isodense to the reminder of the spleen. The medial (internal) lobe of the spleen extends medially displacing the upper pole of left kidney downwards and gently indenting the posterior aspect of the gastric fundus.

**Figure 5.**
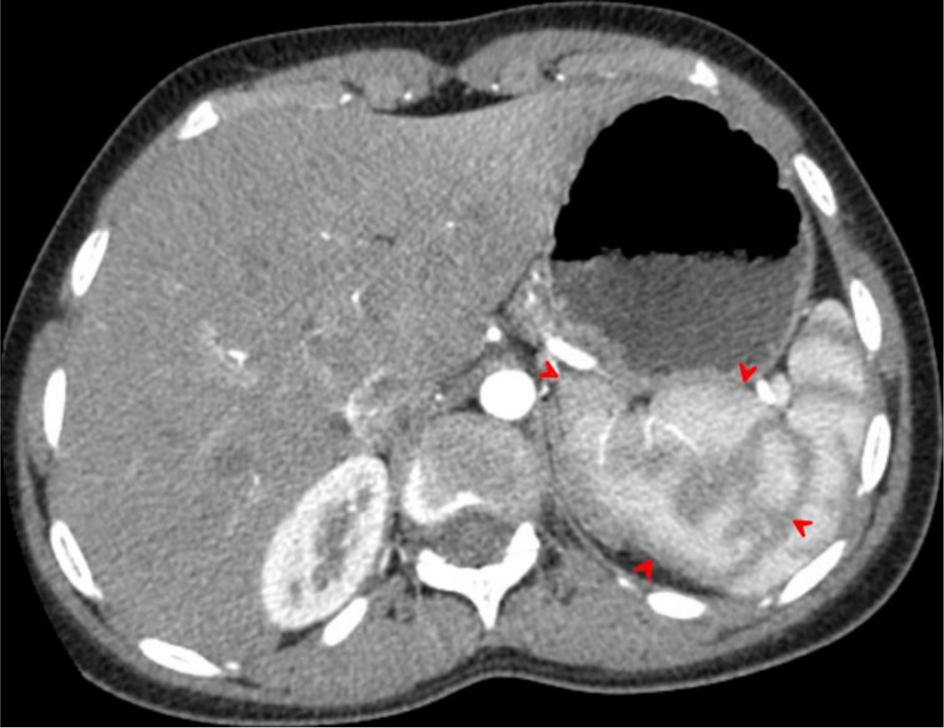
Axial contrast-enhanced CT image acquired during the arterial phase through the upper spleen demonstrates the medial internal lobe of the spleen (red arrowheads) shows the characteristic mottled appearance, extending medially displacing the upper pole of left kidney downwards and gently indenting the posterior aspect of the gastric fundus. The upper pole of the right kidney seen while the upper pole of the left kidney not demonstrated (displaced downwards).

**Figure 6.**
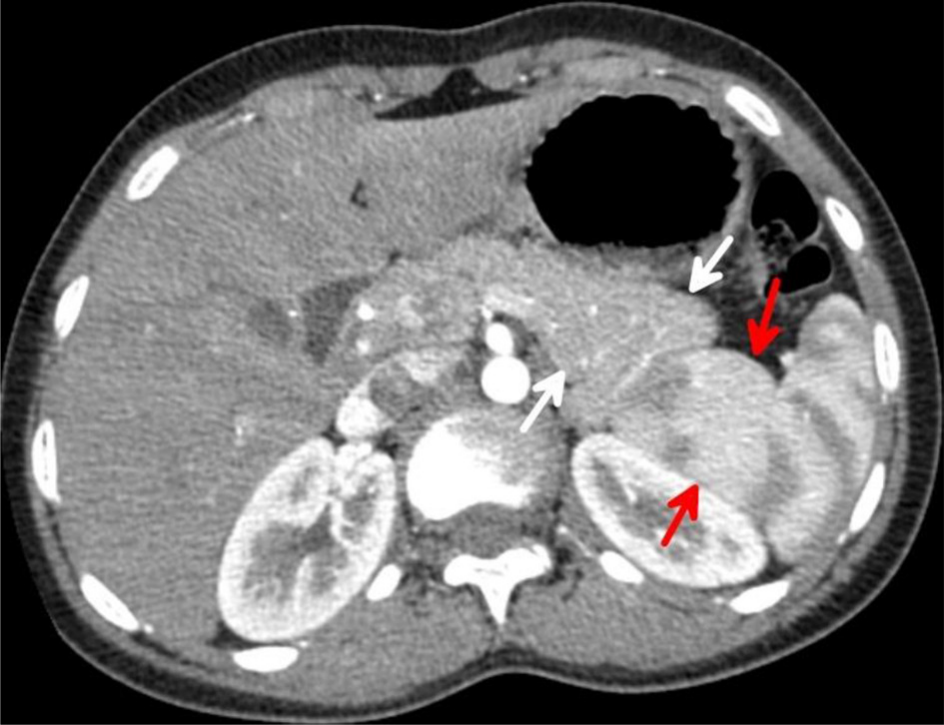
Transverse contrast-enhanced CT images acquired during the arterial phase through the mid-spleen demonstrates the medial internal lobe of the spleen (red arrows) insinuated between the compressed tail of the pancreas (white arrows) and the left kidney. The mottled (zebra lines) enhancement pattern of the spleen during the arterial phase distinguishes the medial (internal) lobe of the spleen and differentiates it from the adjacent tail of the pancreas and upper left kidney.

**Figure 7.**
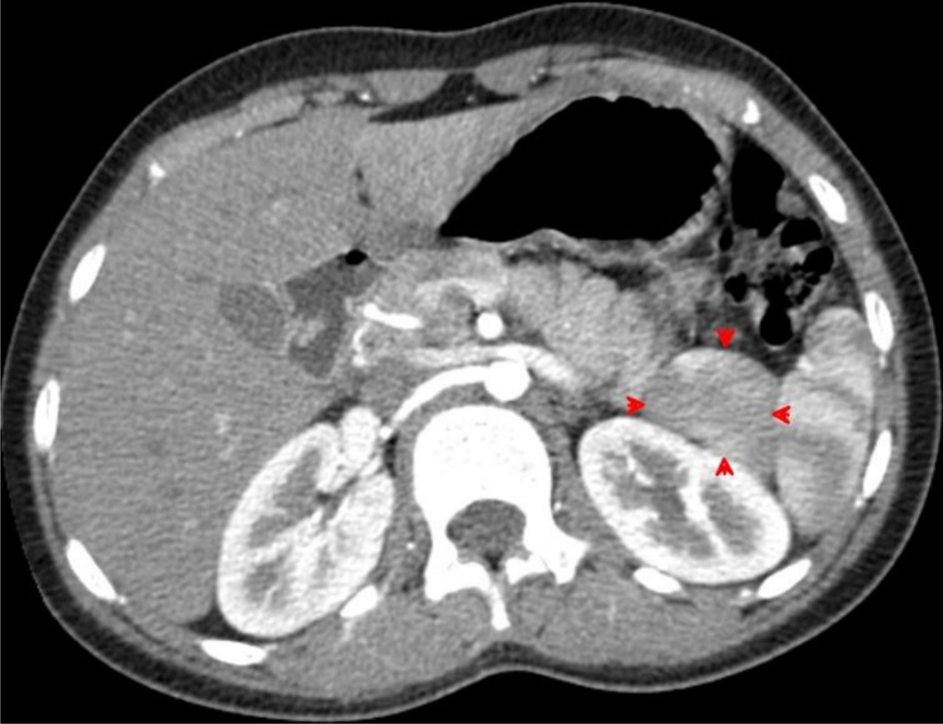
Transverse contrast-enhanced CT images acquired during the arterial phase through the lower spleen demonstrates the medial internal lobe of the spleen (red arrowheads) insinuated between the compressed tail of the pancreas and the left kidney. The mottled (zebra lines) enhancement pattern of the spleen during the arterial phase distinguishes the medial (internal) lobe of the spleen and differentiates it from the adjacent tail of the pancreas and upper left kidney.

**Figure 8.**
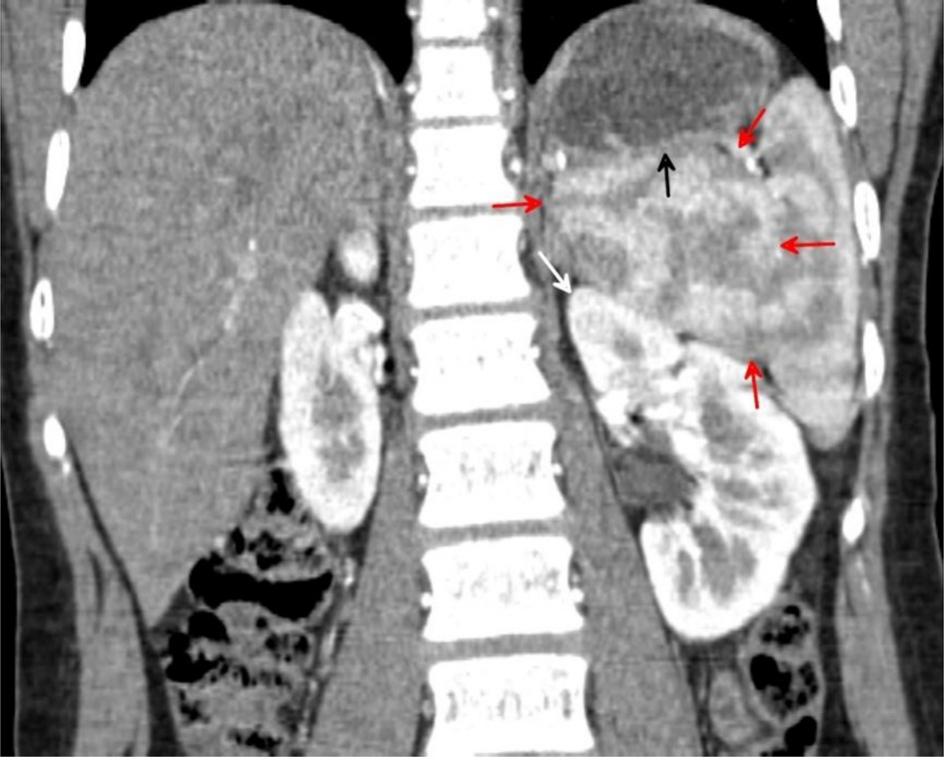
Coronal multiplanar reformation CT image acquired during the arterial phase demonstrates the medial (internal) lobe of the spleen (red arrows), extending medially displacing the upper pole of the left kidney (white arrow) downwards and gently indenting posterior aspect of the gastric fundus (black arrow). The upper pole of the right kidney seen higher than its left counterpart. The medial splenic lobe shows the characteristic mottled appearance of splenic parenchyma on arterial phase.

**Figure 9.**
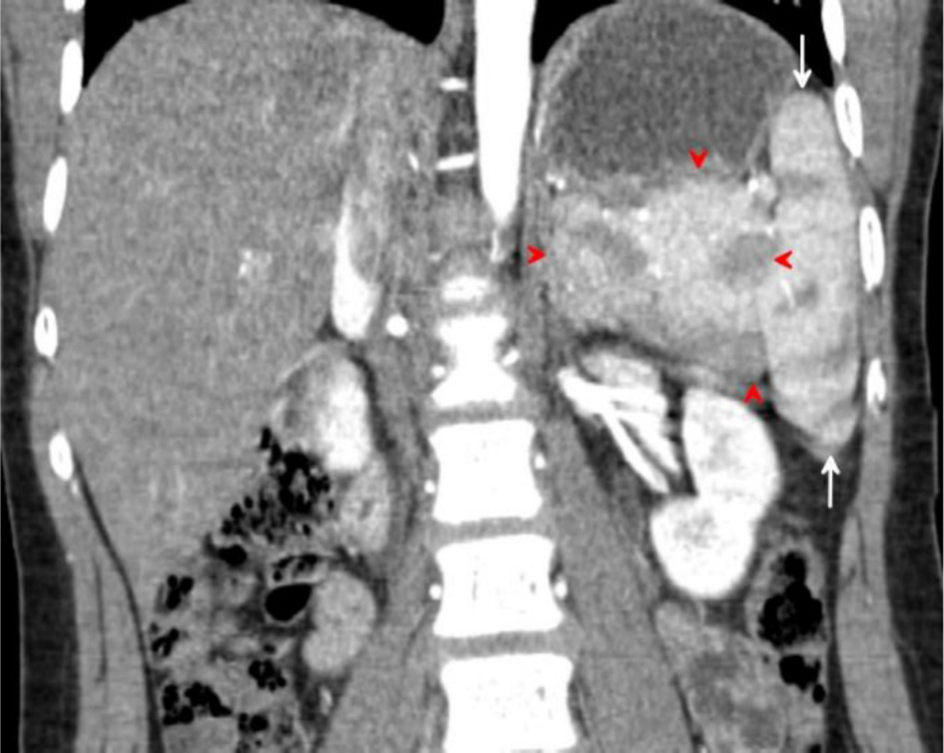
Coronal multiplanar reformation CT image acquired during the arterial phase demonstrates the medial (internal) lobe of the spleen (red arrowheads), extending medially in a transverse manner. The lateral (external) lobe of the spleen (white arrows) attains a lateral and vertical orientation.

**Figure 10.**
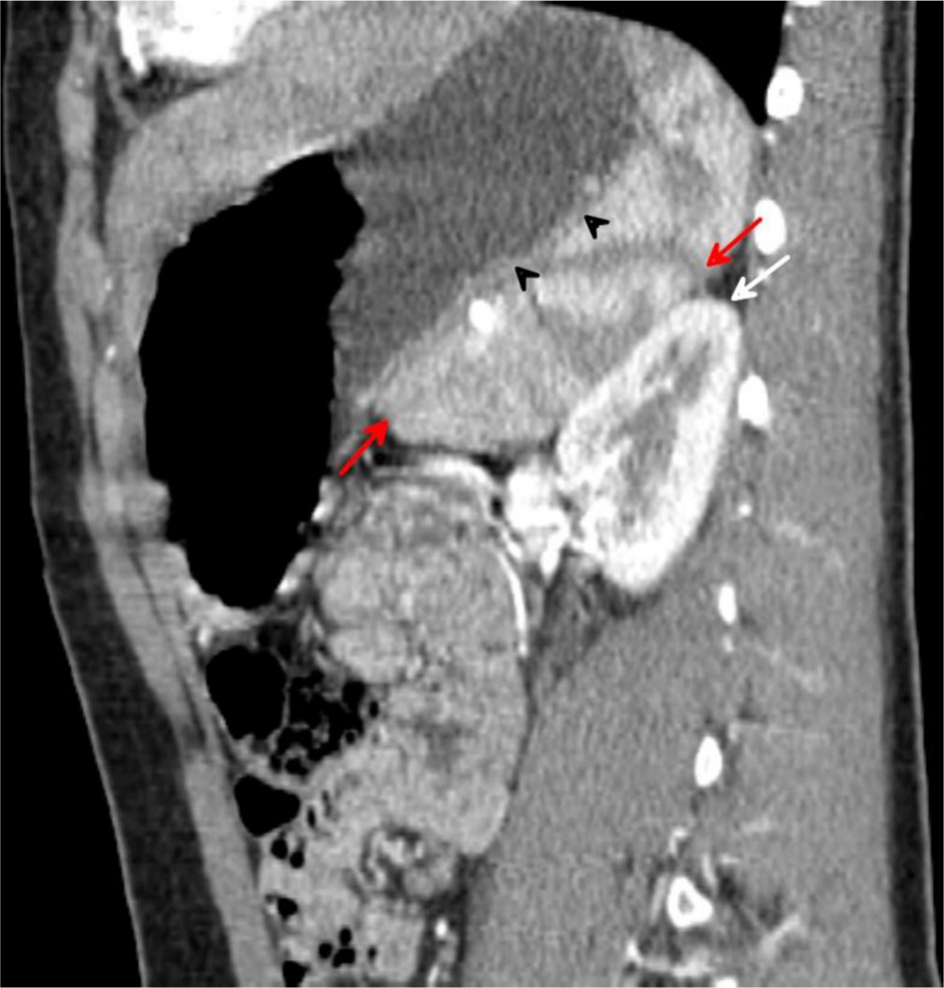
Sagittal multiplanar reformation CT image acquired during the arterial phase demonstrates the medial (internal) lobe of the spleen (red arrows), extending medially displacing the upper pole of the left kidney (white arrow) downwards and gently indenting posterior aspect of the gastric fundus (black arrowheads).

**Figure 11.**
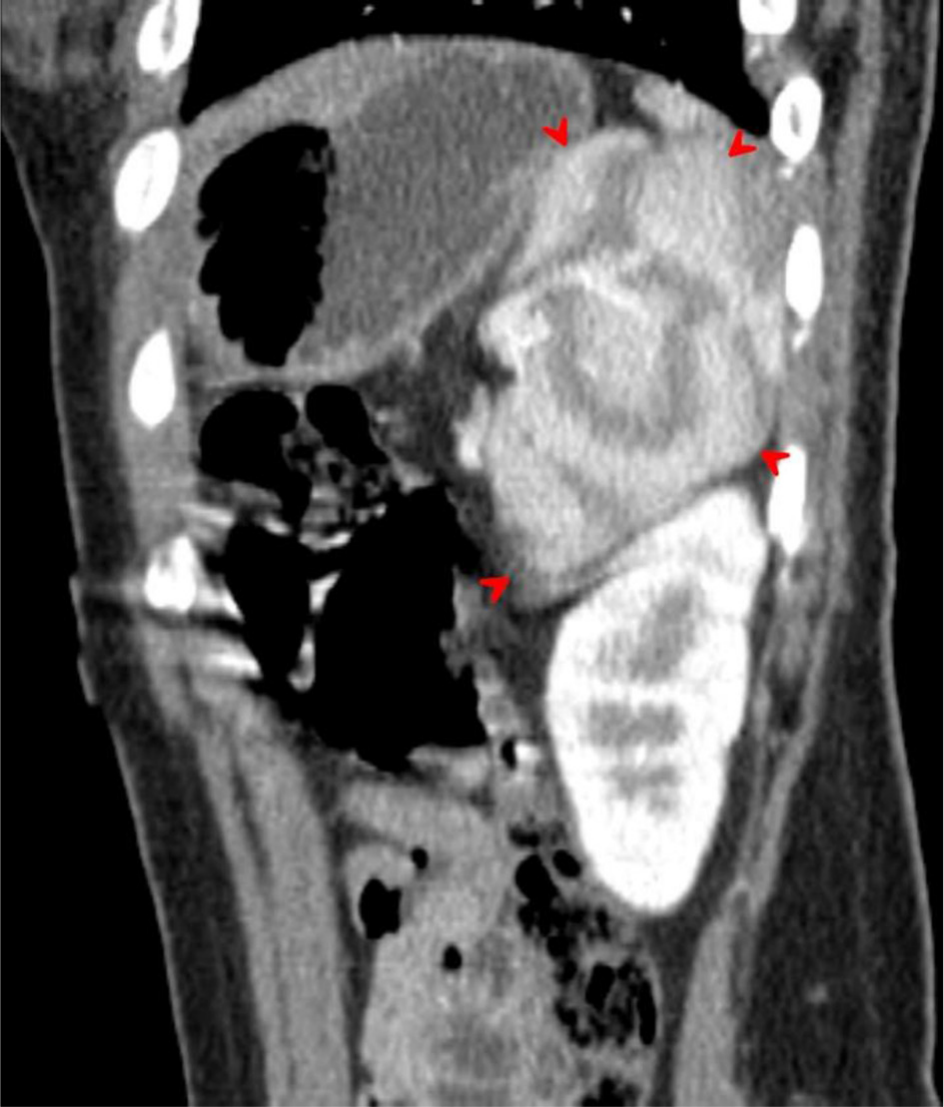
Sagittal multiplanar reformation CT image acquired during the arterial phase demonstrates the medial (internal) lobe of the spleen (red arrowheads), extending medially in a transverse manner, displacing the upper pole of left kidney downwards and gently indenting posterior aspect of the gastric fundus.

The shape of the spleen exhibits a wide variability. On arterial-phase CT images, the spleen typically shows a mottled (i.e. zebra lines) enhancement pattern due to variable flow rates of contrast-enhanced blood through the sinuses of the red pulp. Awareness of this heterogeneous enhancement pattern is crucial, because underlying pathologies or focal lesions may be obscured. On portal venous-phase CT images, healthy splenic parenchyma has a homogenous appearance. Portal venous-phase CT images should be evaluated when searching for and interpreting lesions of the spleen.

The bilobed spleen is an extremely rare morphologic variation of the spleen. An oblique fissure was located longitudinally at the mid-region of the visceral surface of the spleen. The medial (internal) lobe of the spleen extends obliquely medially to compress and slightly displace the tail of the pancreas and left adrenal gland. The medial (internal) splenic lobe significantly displaces the left kidney downwards.

The bilobed spleen should be differentiated from causes of splenomegaly and other splenic solid masses. The bilobed spleen should be differentiated from a wide variety of solid masses at the surrounding region such as pancreatic body and tail solid lesions (neuroendocrine tumours, pancreatic metastases, acinar cell carcinoma, pancreatoblastoma, solitary fibrous tumour, pancreatic hamartoma, serous adenoma, intrapancreatic splenculus). The medial lobe of the bilobed spleen should be differentiated from solid masses arising from the gastric fundus such as exophytic gastrointestinal stromal tumours. It should also be differentiated from neurogenic tumours as peripancreatic paragangliomas. The medial lobe of the bilobed spleen should also be differentiated from the solid masses of the left adrenal gland (myelolipoma, adenoma, pheochromocytoma) and the upper pole of the left kidney (hypernephroma, Wilms). Radiologists and clinicians should make sure that the fissure in a spleen is not misinterpreted as a laceration or rupture in the abdominal trauma setting.

## Conclusions

The present case is an extremely rare morphologic congenital anomaly of the spleen. The spleen is composed of medial (internal) and lateral (external) lobes connected at the hilum. The medial lobe is seen extending medially, compressing and slightly displacing the tail of the pancreas and left adrenal gland and gently indenting the posterior aspect the gastric fundus. The left kidney seen displaced downwards. The lienorenal ligament was not obvious. The medial lobe large enough to compress the tail and body of the pancreas and left adrenal gland gently indents the posterior aspect of gastric fundus and to displace the left kidney downwards. The medial lobe demonstrates the characteristic mottled appearance of splenic parenchyma on arterial-phase CT images. The rarely encountered bilobed spleen could be confused with splenomegaly. Bilobed spleen as reported here might cause misinterpretations as a mass originating from the tail of the pancreas, left adrenal gland or the fundus of the stomach. The mottled (zebra lines) configuration of the splenic parenchyma on post-contrast arterial phase CT is a crucial distinguishing factor.

## Learning points

1. Knowledge of the possible anomalies and different morphologic variants of the spleen is important in order to avoid pitfalls in the interpretation of abdominal imaging studies such as CT and ultrasonography.
2. For this reason, this case report demonstrates an extremely rare variant of the normal spleen which could be confused for splenic or pancreatic space occupying lesions.
3. The recurrent left hypochondrial pain in the current case could be attributed to attempts of strangulation of the vascular pedicle at the splenic hilum with the absence of the lienorenal ligament.
4. No available appreciable data regarding bilobed spleen and its related findings and symptoms in the imaging literature.

## Consent

Written informed consent for the case to be published (including images, case history and data) was obtained from the patient(s) for publication of this case report, including accompanying images.
